# Randomized Prospective Comparison of Two Protocols for Head-up Tilt Testing in Patients with Normal Heart and Recurrent Unexplained Syncope

**DOI:** 10.1016/s0972-6292(16)30689-1

**Published:** 2013-11-15

**Authors:** Mohammad Alasti, Mohammad Hosein Nikoo, Mohommad Hosein Jadbabaei, Masoud Seyedian, Babak Payami, Saman Taghavianpour, Bita Omidvar, Maryam Maghoumizadeh, Nasim Azadi

**Affiliations:** 1 Department of Cardiology, Imam Khomeini Hospital, Ahvaz Jundishapur University of Medical Sciences, Ahvaz, Iran; 2 Department of Cardiology, Kosar Hospital, Shiraz University of Medical Sciences, Shiraz, Iran; 3 Department of Cardiology, Golestan Hospital, Ahvaz Jundishapur University of Medical Sciences, Ahvaz, Iran; 4 Department of Internal Medicine, Golestan Hospital, Ahvaz Jundishapur University of Medical Sciences, Ahvaz, Iran

**Keywords:** Head-up tilt test, Neurally mediated syncope, Unexplained syncope

## Abstract

**Background:**

This randomized study was aimed to compare the diagnostic value of two head-up tilt testing protocols using sublingual nitroglycerin for provocation in patients with recurrent unexplained syncope and normal heart.

**Methods:**

The patients with normal findings in physical examination, electrocardiography and echocardiography were randomly submitted to one of upright tilt test protocols. The only difference between two protocols was that nitroglycerin was administered after a five minute resting phase in supine position during protocol B. We also considered eighty normal persons as the control group.

**Results:**

Out of 290 patients that underwent tilt testing, 132 patients were in group A versus 158 patients in group B. Both groups had an identical distribution of clinical characteristics. Tilt test was positive in 79 patients in group A (25 in passive phase, 54 in active phase) versus 96 patients in group B (43 in passive phase, 53 in active phase). There was no significant difference between results in two groups (P value= 0.127). Forty cases were tested with protocol A and forty underwent tilt testing with protocol B. Tilt test was positive in 4 cases with protocol A versus 3 cases in protocol B. The positive rates of tilt testing with protocol A was 60% while it was 61% in protocol B. The specificity of testing with protocol A was 90% and it was 92.5% in protocol B.

**Conclusions:**

According to our data, adding a period of rest and returning to supine position before nitroglycerin administration had no additional diagnostic yield.

## Introduction

Neurally mediated syncope is the most common cause of syncope at any age and upright tilt testing has been one of the most common tests in assessing the patients with unexplained syncope. [1,2] Many protocols have been proposed for this purpose and the Italian protocol has been suggested as the standard protocol.[3] But honestly, there is no real gold standard test and the results of several studies have shown conflicting results concerning sensitivity and specificity of the test.

Sublingual nitroglycerin has become popular agent for potentiating of head-up tilt tests in adults because of simplicity of use. Nitrates have vasodilatory effect especially on veins and this effect varies in different positions. [4,5]

We hypothesized that the position of the patient during nitroglycerin administration may have some effect on sensitivity and specificity of upright tilt testing. In this study, we decided to compare the positive rate and specificity of two head-up tilt testing protocols using sublingual nitroglycerin as the provocative-agent in patients with normal heart and recurrent unexplained syncope. The only difference between these two protocols was the position of the patient at which nitroglycerin was administered. We hypothesized that this new protocol may have a better specificity than the other protocol.

## Methods

### Study population

We considered all the patients that were referred to Jundishapur University and Shiraz University affiliated hospitals for evaluation of syncope from 1st January 2009 to 31th December 2011. All cases underwent history taking, physical examination, neurologic assessment, 12-lead conventional electrocardiography and transthoracic echocardiography. Exercise stress test was performed only when clinically indicated. The patients who had 1) age of twenty-five to forty-five 2) at least two episodes of syncope 3) normal physical examination including supine and orthostatic blood pressure 4) normal electrocardiography 5) normal echocardiographic study were selected. The patients who had diabetes, autoimmune disease and neurologic disorders were excluded. We also excluded patients with clinically typical neurally mediated syncope, e.g. the patients with situational syncope.

All eligible subjects were randomized via random numbers table and allocated with sealed envelopes to either protocol A or protocol B and one week later, crossed over to the other protocol at the same time of the day. After testing 50 patients in each group, we compared the results in each group. There were no significant differences between the results. Hence, we performed only one upright tilt test in other patients in each group and compared the results of tests in group A versus group B. The main reason of this decision was decreasing of the time and the cost needed for the study.

We also considered eighty healthy cases with normal physical examination, normal 12-lead electrocardiography and transthoracic echocardiography that did not have any episode of syncope in the past as the control group. We select them among medical staff and students in age group of 25-45. Randomly, forty of them underwent upright tilt testing with protocol A and the rest with protocol B.

### Head-up tilt test with nitroglycerin provocation

We used a tilt table with footboard support located in a quiet room. To avoid diurnal autonomic variability interference, we performed all tests between 8 a.m. and 10 a.m. All cardiovascular drugs were withdrawn for at least 5 half-lives before the study. All cases had been fasting for at least four hours. Antecubital venous cannulation was performed before resting phase and the electrocardiogram and blood pressure were measured and recorded by the Task Force monitor (CN systems, Graz, Austeria). Sublingual spray nitroglycerin (Nitromint, EGIS Pharmaceuticals PLC, Budapest, Hungary) was used as the provocative agent. Oral spray was chosen because its action is faster and its absorption is better.

We used two head-up tilt test protocols which were different in active phase.

*Protocol A*: Resting phase in supine position for 20 min after venous cannulation, Drug-free 70º tilt for 45 min, Active 70º tilt phase (after 400 μg sublingual nitroglycerin administrations) for 15 min. This protocol is the protocol that routinely is used at Jundishapur University of Medical Sciences.

*Protocol B*: Resting phase in supine position for 20 min after venous cannulation, Drug-free 70º tilt for 45 min, Resting phase in supine position for 5 min, Active 70º tilt phase (after 400 μg sublingual nitroglycerin administration) for 15 min.

The tilt table was lowered when a positive result was developed or when the test ended according to the protocol.

### Definitions

We defined syncope as sudden and transient loss of consciousness and postural tone followed by spontaneous recovery. Head-up tilt test was considered positive if syncope was reproduced in association with hypotension, bradycardia or both.[5]

### Ethics

The study protocol was approved by ethics committee of Jundishapur University of Medical Sciences. All patients provided written informed consent.

### Statistical analysis

Continuous data were expressed as mean ± standard deviation values. Chi-square test and student T-test were used to compare groups. A P value less than 0.05 was considered to be statistically significant.

## Results

During the first part of the study, fifty patients underwent upright tilt test in each group and the test was repeated with the other protocol one week later. The demographic and clinical characteristics of the patients in these two groups and the results of two separate upright tilt tests in each group are shown in Table 1 and Table 2. There was no significant difference between demographic and clinical characteristics of the patients in both groups. The results of repeating upright tilt test were not significantly different from the results of first test. After that we decided to perform only one test on other patients in each group to decrease the time and the budget needed for the study.

Overall two hundred ninety patients were enrolled. Head-up tilt test was performed in one hundred thirty-two patients with protocol A and one hundred fifty-eight patients with protocol B. The demographic and clinical characteristics of the patients in group A and group B are shown in Table 3. There was no significant difference between two groups except in corrected QT interval, although both were within normal range.

Forty control cases underwent head-up tilt testing with protocol A. The mean age of this group was 29.9±10.2 years and the male/female ratio was 20(50%)/20(50%). On the other hand, forty control cases underwent tilt testing with protocol B. The mean age of the group was 29.3±11.1 years and the male/female ratio was 22(55%)/18(45%). All the echocardiographic and electrocardiographic parameters were within normal limits.

No serious side effect was observed during the study except for mild headaches in twenty-nine patients and six controls.

The results of tilt testing in group A and group B are shown and compared in Table 4.

Among control group, four cases in protocol A group and three cases in protocol B group had positive results. The syncope occurred in all of these persons after nitroglycerin administration.

Because we did not have a real gold standard to diagnose the vasovagal syncope, we considered comparison of the positive rate of tests with different protocols instead of their sensitivity. The positive rate of protocol A was 60% and the positive rate of protocol B was 61%. The specificity of protocol A was 90% and on the other hand, the specificity of protocol B was 92.5%.

To compare the effect of patient's position at the time of drug administration on test results in active phase, we excluded the patients with positive result in passive phase of tilt test and repeated the comparison. Out of 107 patients in group A, 53 patients had negative results and 54 patients had positive results in active phase and out of 115 patients in group B, 62 patients had negative results and 53 patients had positive results. The difference between two groups was no significant (P value: 0.652). According to these results, the positive rate of protocol A in active phase was 50% and the positive rate of protocol B was 46%.

We compared the patients' characteristics in cases with positive upright tilt test with the cases without positive results (Table 5). The only difference was the history of traumatic syncope that was more prevalent in patients with positive upright tilt test result. Age, gender, time from the first syncope, time from the last syncope and number of syncopal attacks did not have any relation with positive upright tilt test results.

We followed all the patients for one year. No arrhythmia or pacemaker implantation had been reported.

## Discussion

Neurally mediated syncope is the most frequent cause of syncope, particularly in patients without structural heart disease.[1] The real mechanism of neurally mediated syncope is still a matter of discussion. It is considered that it can be initiated by venous pooling, with a subsequent reduced blood volume and increased sympathetic activity that in the susceptible persons can result in paradoxical activation of the ventricular mechanoreceptors with inhibitory afferents to cardiovascular brainstem centers, which are ultimately responsible for the triggering of bradycardia or hypotension or both.[1] Other factors such as neurohumoral activation and higher central nervous centers could also contribute.[1]

Head-up tilt testing is the most popular technique for the detection of this common type of syncope although there are wide variations in protocols and provocative agents used in different centers. [2-14] While upright tilt test with the Italian protocol has been proposed as the standard method of upright tilt test in vasovagal syncope evaluation,[3] there is not a real gold standard test for diagnosis of neurally mediated syncope.

Sublingual nitroglycerin has become the most common agent for potentiating of head- up tilt testing in adults because of its convenience and safety. Because of its potent vasodilatory effect in the capacity vessels, nitrates increase venous pooling already enhanced by the upright posture and can increase the sensitivity of test.[1] Nitrates are lipid soluble and readily cross the blood-to-brain barrier, so nitrates may have a central role in tilt test potentiation.[15] Nitrate potentiated tilt testing has been used in many studies with various protocols. In most protocols sublingual nitrate was administered in the standing position without returning to a supine position. [15] We hypothesized that the total amount of orthostatic stress was less in protocols that lower the table before drug administration. On the other hand, as mentioned before, nitrates may potentiate the test with central role that is not related to the position of the patient. The present study was designed to determine the influence of patient's position before nitrate administration on test results. As a matter of fact, we wanted to compare the positive rate of a protocol with probable better specificity (protocol B) with the routine protocol (protocol A). The only difference between two protocols we used was the position of patient before drug administration. To best of our knowledge, a comparison between diagnostic yields of these two protocols of tilts had never been performed.

According to our data, reproducibility of the test results was excellent compared to other studies that showed a reproducibility rate of 80% for positive results.[2]

In our study, the positive rates in protocol A were 60% versus 61% in protocol B which were similar to previous studies (61-69%).[3] The specificity of upright tilt testing with the common protocols has been reported 92-94%.3 According to this study, the specificity of tilt testing with protocol A was 90%. On the other hand, the specificity of tilt testing with protocol B was 92.5% that was not significantly different from the protocol A.

We also measured the positive test rates in active phase to compare the two protocols after drug provocation. The positive rate in upright tilt testing with protocol A was 50%, while the positive rate in protocol B was 46%. It means that adding a period of rest to the common tilt testing protocol not only do not increase the specificity of test but also may decrease its sensitivity.

In our study, sublingual nitroglycerine was safe and well tolerated. In fact, after drug administration, the test was complicated by headache in thirty-five patients (9%), which nevertheless did not request test interruption.

## Study limitations

One major limitation of the present study is that the mean age of our patients was low and the results cannot be applied to all patients with vasovagal syncope. The relatively small number of patients in each subgroup is another limitation for an adequate statistical analysis.

## Conclusion

According to our data, adding a period of rest to head-up tilt testing protocol and returning to supine position before nitroglycerin administration had no additional diagnostic yield.

## Figures and Tables

**Table 1:**
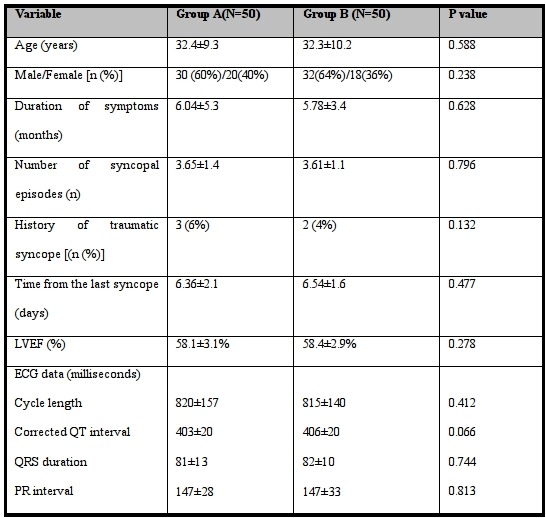
Baseline characteristics of the patients in groupA versus the patients in group B during the first part of the study

**Table 2:**
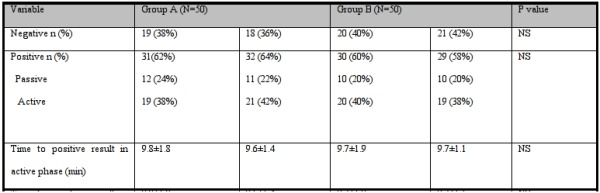
Comparison of head-up tilt results in the patients in groupA versus the patients in group B during the first part of the study

NS: Not Significant

**Table 3:**
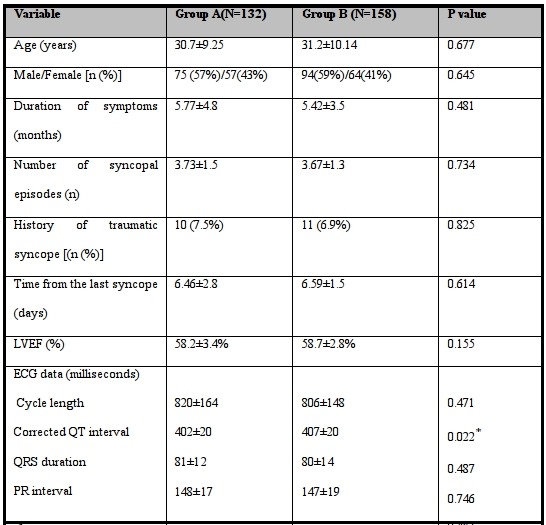
Baseline characteristics of patients in group A versus group B

* Statistically significant difference between two groups (P<0.05).

**Table 4:**
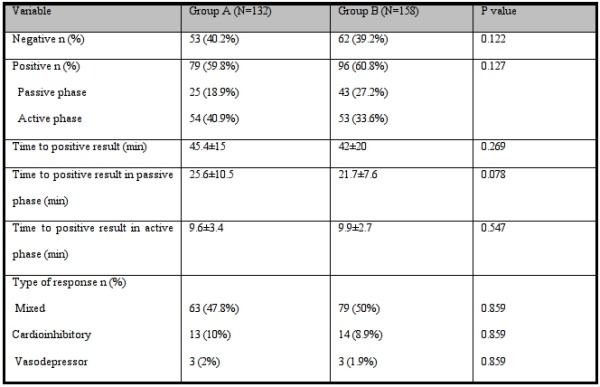
Comparison of head-up tilt results in group A versus group B

**Table 5:**
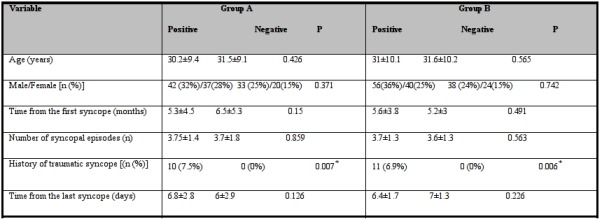
The relation between results and clinical characteristics of patients in group A and group B

* Statistically significant difference between two groups (P<0.05).
